# Rubber-like and Antifouling Poly(trimethylene carbonate-ethylphosphonate) Copolymers with Tunable Hydrolysis

**DOI:** 10.1021/acsami.4c21079

**Published:** 2025-04-15

**Authors:** Timo Rheinberger, Marc J. K. Ankone, Dirk W. Grijpma, Frederik R. Wurm

**Affiliations:** † Sustainable Polymer Chemistry (SPC), Department of Molecules and Materials, MESA+ Institute for Nanotechnology, Faculty of Science and Technology, University of Twente, P.O. Box 217, 7500 AE Enschede, The Netherlands; ‡ Department of Advanced Organ Bioengineering and Therapeutics (AOT), Faculty of Science and Technology, University of Twente, Enschede 7522 NB, The Netherlands

**Keywords:** microstructure, polycarbonate, material properties, enzymatic degradation, stealth properties

## Abstract

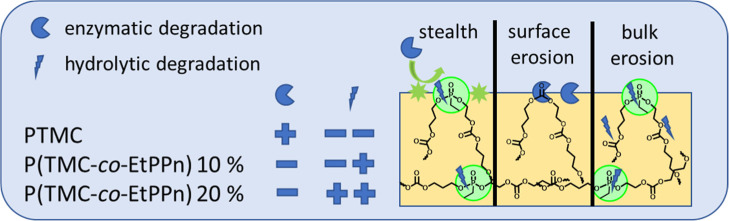

Controlling the degradation and cell interaction of polymer materials is vital for numerous applications. Transitioning from enzymatic to nonenzymatic hydrolysis offers precise control over degradation processes. In this study, we synthesized high molar mass poly(trimethylene carbonate) (PTMC)–polyphosphonate copolymers to achieve distinctive antifouling and controlled degradation properties. 2-Ethyl-2-oxo-1,3,2-dioxaphospholane (EtPPn) is copolymerized with trimethylene carbonate (TMC) to random P(TMC-*co*-EtPPn) copolymers through ring-opening copolymerization, utilizing Sn(Oct)_2_ as the catalyst. Copolymers with molar masses reaching up to *M*_n_ = 218 kg/mol and molar mass dispersities of *D̵* < 1.9 are obtained. To maintain hydrophobicity, 10 and 20 mol % of hydrophilic phosphonate units are incorporated into PTMC-copolymers. While copolymers with 10 mol % EtPPn display mechanical properties akin to the homopolymer PTMC, a deviation in elongation at break and yield strength results when 20 mol % EtPPN is incorporated. PTMC–PPE copolymers demonstrate antifouling behavior, i.e., cell repulsion for human mesenchymal stem cells (hMSCs) and inhibited enzymatic degradation by lipase in contrast to PTMC-homopolymers. Conversely, P(TMC-*co*-EtPPn) undergo abiotic hydrolytic degradation with hydrolysis rates increasing with increasing phosphonate contents. In conclusion, copolymerization with EtPPn enables the switch from enzymatic PTMC degradation to adjustable hydrolytic degradation, offering controlled stabilities of such copolymers in the desired applications.

## Introduction

For biodegradable polymers, like aliphatic polycarbonates and polyesters, adjusting of the hydrolysis kinetics is achieved by changing the polymers’ hydrophilicity by the chemical structure of the monomer or by copolymerization with hydrophilic monomers.^1,2^ A prominent example is poly(lactide-*co*-glycolide) (PLGA), in which the more hydrophilic glycolic acid (GA) units increase the hydrolysis rates compared to polylactic acid (PLA).^3,4^ Similarly, one could expect that hydrophilic phosphoester units would accelerate hydrolysis in random copolymers.^5^ Previous studies by us and others proved an accelerated hydrolysis of PLA-*co*-PPE copolymers, when special breaking points were installed.^6,7^ Copolymerization of cyclic phosphoesters with cyclic carbonate has not been studied in depth.^8^ In addition, to date the enzymatic degradation of such copolymers had not been investigated; especially as polyphosphoesters are known to exhibit antifouling properties, the latter might influence biodegradation behavior.^9−11^

Hence, this work explores the antifouling properties and enzymatic hydrolysis of poly(trimethylene carbonate-*co*-ethyl ethylene phosphonate) copolymers and compares it to their abiotic hydrolysis.

Poly(trimethylene carbonate) (PTMC) has gained significant attention in the field of medical applications, being utilized in various forms such as copolymers or blends.^12−14^ PTMC is widely used in medical products and biomedical research as drug delivery and tissue engineering, exemplified by (Maxon and Inion CPS).^15^ Achieving desired mechanical and biological properties often involves copolymerization or cross-linking.^16,17^ However, there can be challenges in balancing the mechanical properties and degradability of these materials. To address these issues, the PPE platform offers versatile strategies, providing adjustable degradation properties as both homopolymers and comonomers in common polymer materials.^6,11,18^ To tailor the mechanical and degradation characteristics of PTMC, it is often copolymerized or blended with other biocompatible polymers such as poly(ε-caprolactone) (PCL), polylactide (PLA), or polyglycolide (PGA).^19^ Copolymerization of the cyclic carbonate monomer can be catalyzed by stannous octanoate (Sn(Oct)_2_), which facilitates the formation of high-molecular-weight polymers, thereby influencing the mechanical properties.^20,21^

In the context of medical applications, the interaction between implants or polymer surfaces and the biological environment is of utmost importance. Both PTMC and PPEs are known for their biocompatibility.^22−24^ While PTMC is commonly used in soft tissue engineering, its cellular interaction is considered moderate.^23^ In contrast, PPEs exhibit unique stealth properties and biomolecule repulsion.^11,25,26^ Therefore, copolymers of PTMC and PPEs hold the potential to exhibit novel and interesting properties when in contact with organic matter.

It is worth noting that PTMC biodegrades following mainly a surface erosion mechanism under enzymatic degradation, while only minimal nonenzymatic hydrolysis occurs at physiological pH.^27^ This surface erosion mechanism ensures that favorable mechanical properties are retained throughout the degradation process. In contrast, hydrophilic PPEs undergo rapid hydrolysis and have been shown to accelerate the degradation of common polymer materials such as polylactic acid (PLA).^6^ Therefore, PTMC–PPE copolymers are expected to exhibit an intriguing degradation behavior depending on the comonomer ratio. To achieve adjustable degradation of the polymer itself, the formation of random copolymers is necessary. Previous research conducted by our team has shown that copolymerization of trimethylene carbonate (TMC) and phosphoester with Sn(Oct)_2_ in bulk leads to random copolymers through transesterification.^8^

In this study, we synthesized high molar mass P(TMC-*co*-EtPPn) copolymers and investigated their structural properties, mechanical performance, and their stability in the presence of enzymatic or hydrolytic conditions. These findings contribute to a deeper understanding of the potential applications of P(TMC-*co*-EtPPn) copolymers in the field of medical polymer materials.

## Results and Discussion

### Polymerization and Molecular Characterization

**Scheme 1:**
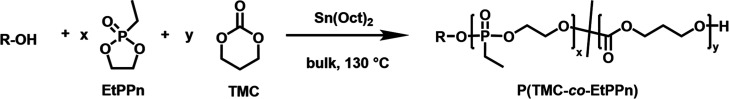
Copolymerization of 2-Ethyl-2-oxo-1,3,2-dioxaphospholane (EtPPn) and Trimethylene Carbonate (TMC) Using Sn(Oct)_2_ as a Catalyst in Bulk at 130 °C (Note That No Exogenous Alcohol was Added as the Initiator)

Copolymers of TMC and 2-ethyl-2-oxo-1,3,2-dioxaphospholane (EtPPn) were synthesized in the bulk with Sn(Oct)_2_ as the catalyst. In order to get PTMC with mechanical properties of interest, a molar mass with an *M*_n_ higher than 90 K g/mol is necessary, to be more than 30 times above the entanglement molar mass.^20^ To obtain that high molar mass, the initiating species are formed in situ from hydroxy impurities in the Sn(Oct)_2_ and monomers.^20^ The TMC monomer was used as received, EtPPn was freshly distilled before use to achieve a sufficient purity, and polymers with *M*_n_ up to 218 K g/mol were synthesized (Scheme 1, Table 1).

**Table 1:** Summarized Properties of the Synthesized Copolymers P(EtPPn-*co*-TMC)^a^

#	polymer	TMC: EtPPn^b^		*M*_n_^c^ (kg mol^–1^)	*M*_w_^c^ (kg mol^–1^)	*D̵*^c^	*T*_g_^d^ (°C)
		initial ratio	final comp				
**1a**	PTMC	100:0	100:0	90	160	1.75	–16
**2**	P(TMC-*co*-EtPPn)	90:10	90:10	218	364	1.67	–20
**3**	P(TMC-*co*-EtPPn)	80:20	77:23	175	324	1.85	–29

a The polymerization was conducted at 130 °C for 26 h in bulk, catalyzed with Sn(Oct)_2_.

b Determined from ^1^H NMR spectroscopy.

c Determined from gel permeation chromatography (GPC) measurements.

d Derived from the second heating curve in differential scanning calorimetry (DSC) measurements (10 °C/min).

**Figure 1:**
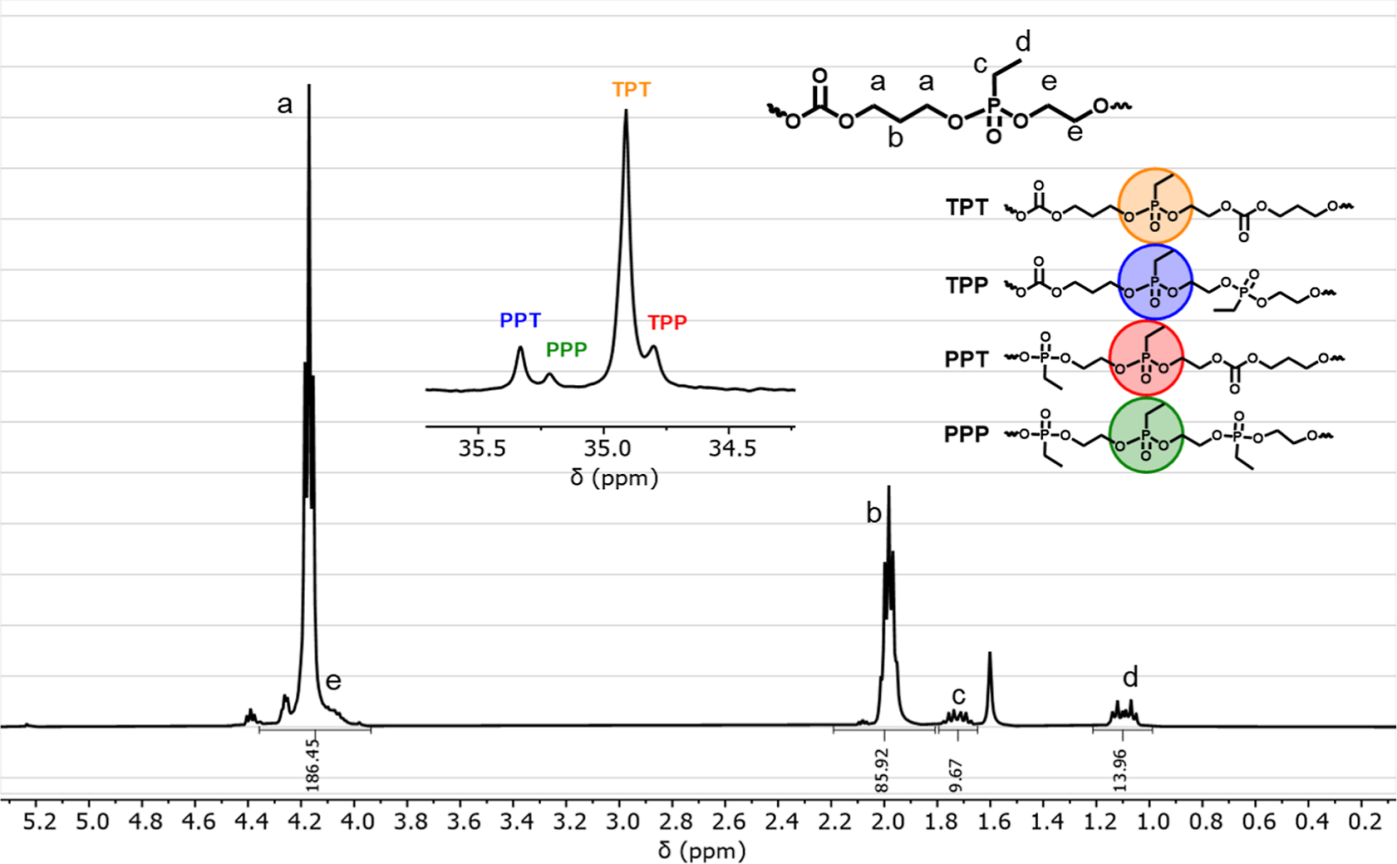
^1^H NMR (400 MHz, 298 K, CDCl_3_) and inset ^31^P{H} NMR (161 MHz, 298 K, CDCl_3_) spectra of polymer **2** P(TMC-*co*-EtPPn) 10% polymerized in bulk at 130 °C.

The synthesized homo- and copolymers targeted with 10 and 20 mol % of EtPPn exhibited unimodal molar mass distributions of *D̵* < 1.9 as determined by GPC, which is in the usual regime for PTMC-homopolymers synthesized in bulk using Sn(Oct)_2_ as a catalyst under these conditions (cf. Figure S1). As comparison, for copolymers of TMC with ε-caprolactone or lactide higher molar mass dispersities were reported.^16,28^ The comonomer ratio in P(TMC-*co*-EtPPn) was determined from ^1^H NMR spectra to be 10 mol % for **2** and 23 mol % for **3**, by comparing the integrals of the PTMC backbone (at ca. 2 ppm) with the side-chain resonances of the phosphonate units at 1.8 ppm (methylene) or at 1.2 ppm (methyl). The ^31^P NMR spectra of P(TMC-*co*-EtPPn) confirmed the randomness of the polymers, showing the expected triad sequences of the monomer units (Figures 1, S2, and S3): ^31^P NMR shows four different resonances for the possible triad sequences with the TPT triad as maximum (inset in Figure 1).

**Figure 2:**
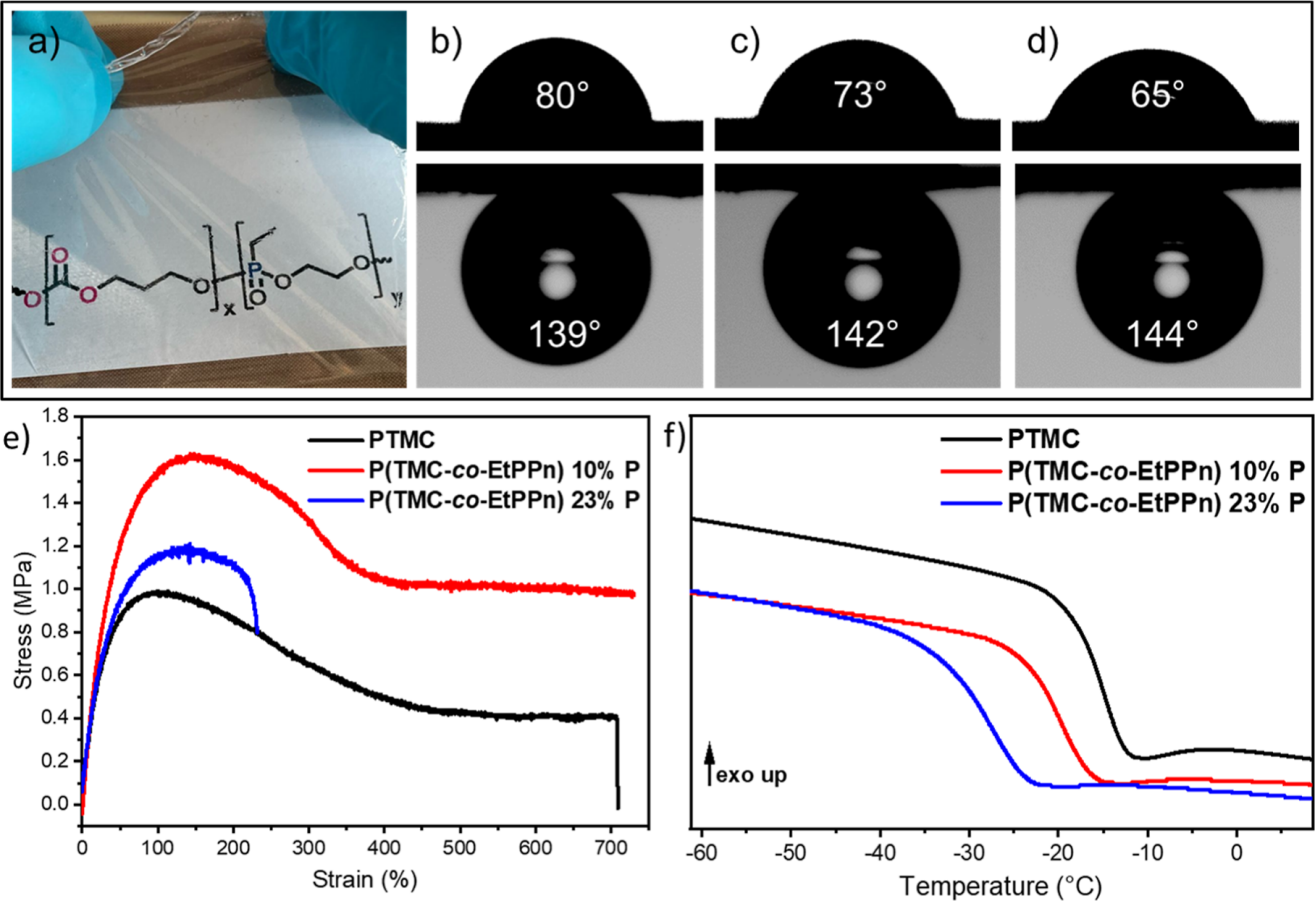
(a) Photograph of the elastic, solvent cast film from P(TMC-*co*-EtPPn (with 10 mol % EtPPn). Contact angle measurements of a water droplet against air (upper picture) and air bubble against water (lower picture) of (b) PTMC (c) P(TMC-*co*-EtPPn) (with 10 mol % EtPPn), and (d) P(TMC-*co*-EtPPn) (with 23 mol % EtPPn). (e) Tensile tests measured on a tensile testing machine; shown is a representative stress strain curve of homo PTMC and the copolymers, (f) DSC thermograms of the polymers; shown is the second heating ramp (10 K/min).

The polymers were solvent cast into films of 0.2 mm thickness (Figure 2) and obtained as colorless and translucent films; these films were used for all further material characterizations. The films showed an increasing hydrophilic surface character for the increasing EtPPn content in the copolymers, indicated by contact angle measurements. The measurement was performed both on a dry film and 1 mL of pure water was deposited on the surface; for PTMC a contact angle of 80° was observed, while the copolymers exhibited increased hydrophilicity (73° for 10 mol % PPE and 65° for 23 mol % PPE). The contact angles in the swollen state (after 5 days immersion in water) were measured in water upside down, and a 1 mL air bubble was deposited on the polymer surface (Figure 2).

### Thermal and Mechanical Properties

All prepared polymers were investigated by dynamic scanning calorimetry (DSC) showing amorphous behavior with a single glass transition temperature between −16 and −29 °C depending on the comonomer ratio (cf. Table 1). The *T*_g_ of the homopolymer PTMC was measured to be −16 °C, in accordance to the literature,^20^ while the PE-containing copolymers exhibited lower glass transition temperatures of −20 (for 10%) or −29 (for 23%). As the homopolymer of PEtPPn exhibits a glass transition temperature of ca. −45 °C, similar to other PPEs prepared by ROP of dioxaphospholanes,^29^ a decreasing *T*_g_ was expected, which was similar to the calculated values according to the Fox equation (−19 °C and −23 °C).^30^

**Figure 3:**
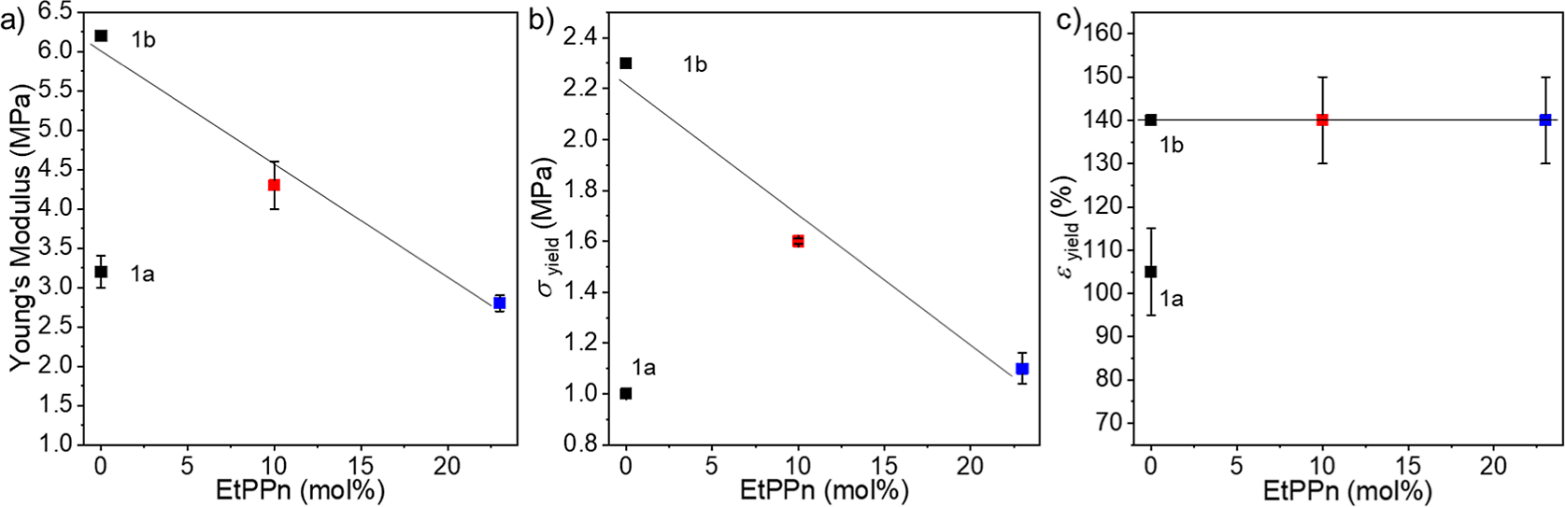
Young’s Modulus (a), stress at yield (*s*_yield_) (b), and elongation at yield (*e*_yield_) (c) in correlation with the content of EtPPn-comonomer in TMC copolymers (results are shown as mean and standard error of the mean, line as guidance for the eye).

Mechanical properties of the different polymers were determined by tensile tests; the observed data are summarized in Figure 2 and Table S1. Since the mechanical properties depend on the molecular weight and the herein used PTMC **1a** had a lower *M*_n_, which resulted in less strength, we compared our copolymer data to literature values provided by Pêgo et al. (called 1b in Figure 3).^20^ For **2,** with 10 mol % EtPPn, the mechanical properties remain similar to those of homo PTMC, in terms of E-modulus, yield strength, and elongation at break. The stress–strain curve from homo PTMC **1a** and P(TMC-*co*-EtPPn) 10% have the same shape, which means that the mechanical properties of PTMC remain similar for less than 10% EtPPn comonomer (Figure 2a). In contrast, if the EtPPn content is 20%, the strength of the material decreases and the elongation at break is less than half. Young’s modulus and stress at yield show a decreasing trend for increasing phosphorus content. The values for the polymer containing 20% phosphorus are comparable to the low molar mass PTMC homopolymer **1a** (Figure 3 a,b). Interestingly, for elongation at yield (Figure 3c) the value for 10% EtPPn is even higher than for the homo polymer, making the polymer more rubber like (Table S1 summarizes all data).

### Cell Interactions

**Figure 4:**
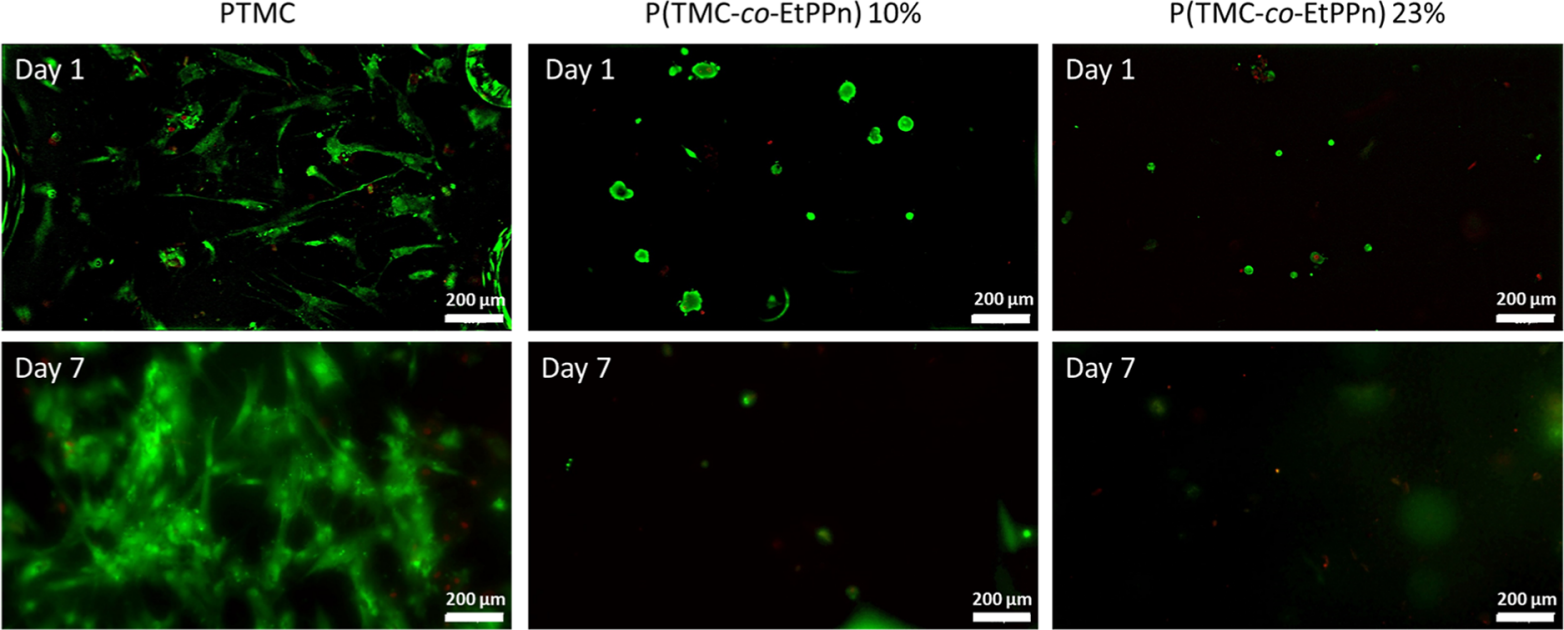
Human mesenchymal stem cells (hMSCs) were cultured on the polymer films; interactions with polymer films. Live/dead staining (green/red, respectively) of hMSCs cultured for 1 and 7 days on the polymer films. Magnification 6.3×; scale bar 200 mm.

To determine the cell interactions of the synthesized polymers, human mesenchymal stem cells (hMSCs) were cultured on the polymer films. Cells were analyzed with fluorescent microscopy labeled with live/dead staining. The cells were clearly spreading on the homo PTMC and growing within 7 days (Figure 4). Only 10% of EtPPn in the copolymer was enough to have a low cell interaction, and in Figure 4, after 1 day the cells are visible as spots almost without spreading. Due to the low attraction most of the cells are washed away during media exchange, therefore after 7 days almost no cells are visible on the polymer surface anymore. For P(TMC-*co*-EtPPn) 20% the cell interaction is more cell repulsion; after 1 day some small cell dots are visible, while after 7 days all cells were detached and washed away from the surface (Figure 4). This shows that for only 10% of EtPPn incorporation the polymer film show stealth effect in terms of cell repulsion.

### Abiotic and Enzymatic Hydrolysis of PTMC and P(TMC-*co*-EtPPn)

#### Abiotic Hydrolysis

Copolymerization is a general strategy to adjust hydrolysis kinetics, commonly achieved by tuning the hydrophilic to hydrophobic ratio in the polymers’ structure.^3,4^ As a hydrophobic polymer, PTMC is relatively robust against nonenzymatic hydrolysis, so at pH values between 1 and 13 no hydrolytic degradation was observed over a period of 8 weeks.^27^ In contrast, the water-soluble PEtPPn was reported to undergo faster hydrolysis with a *t*_1/2_ of ca. 130 h at pH 8, while enzymatic degradation was not reported to date.^18^ In the presence of enzymes, like lipase, PTMC is known to hydrolyze following mainly a surface erosion mechanism.^27^

**Figure 5:**
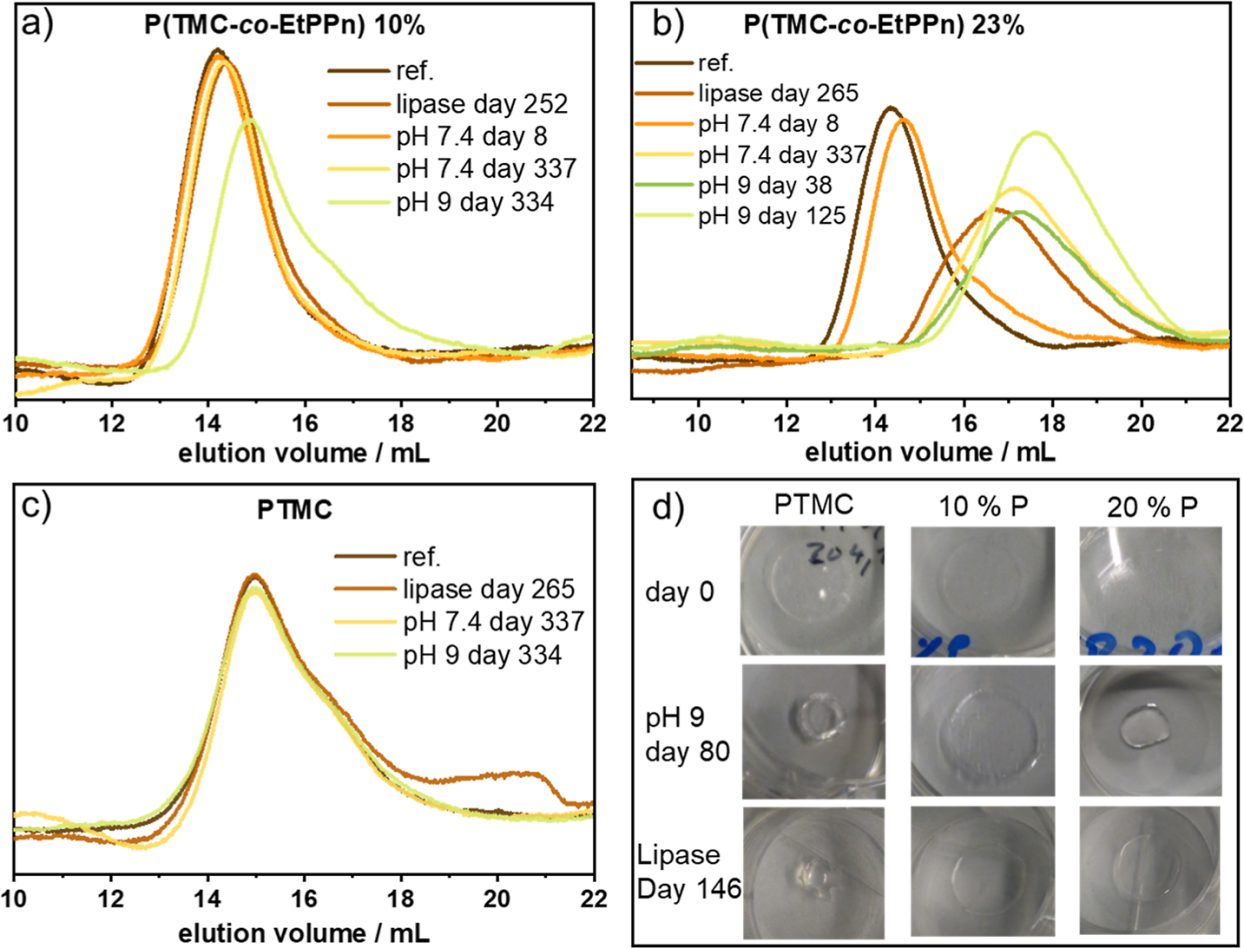
Abiotic and enzymatic hydrolysis of PTMC and P(TMC-*co*-EtPPn) followed visually and by GPC. (a) polymer **2**, (b) polymer **3**, and (c) polymer **1a** before and after hydrolysis using different conditions at certain time points; (d) photographs of the polymer films before hydrolysis, after 80 days at pH 9, and after 146 days in lipase solution.

Here, we studied the abiotic hydrolysis of the (co)polymer films at pH 7.4 (in PBS) and at pH 9 (in a borax buffer) to investigate the effect of the hydrophilic and more labile phosphonate linkages on the overall hydrolysis. The degree of hydrolysis was determined by mass loss and GPC measurements. As reported before,^27^ PTMC also showed barely any signs of hydrolysis under our conditions over a period of one year, indicated by no shift of the molar mass distribution in GPC elugrams and only one percent of detected mass loss, which is in the margin of the weighing error (Figures 5 and S4). The film prepared from the copolymer containing 10 mol % EtPPn (**2**) also showed no mass loss at pH = 7.4 (after 1 year), while less than 4 weight % mass loss at pH = 9 within one year was detected (Figures 5 and S5). The GPC traces during the hydrolysis experiment of **2** in PBS showed almost no shift, indicating no hydrolysis of phosphonate linkages in the bulk of the specimen occurred. In contrast, at pH = 9, molar mass distributions (in GPC) shifted slightly to higher elution times indicating bulk erosion (Figures 5a and S5). For sample **3**, with 23 mol % of EtPPn comonomer content, both at pH = 7.4 and pH = 9 clear shifts of the molar mass distribution in the GPC elugrams were detected (Figure 5b). Additionally, the polymer film visually degraded into a liquid droplet (cf. Figure S7), which caused floating and the loss of the polymer samples (note: it was no longer possible to follow the degradation experiment at pH 9 and it was terminated after 125 days).

#### Enzymatic Degradation

**Figure 6:**
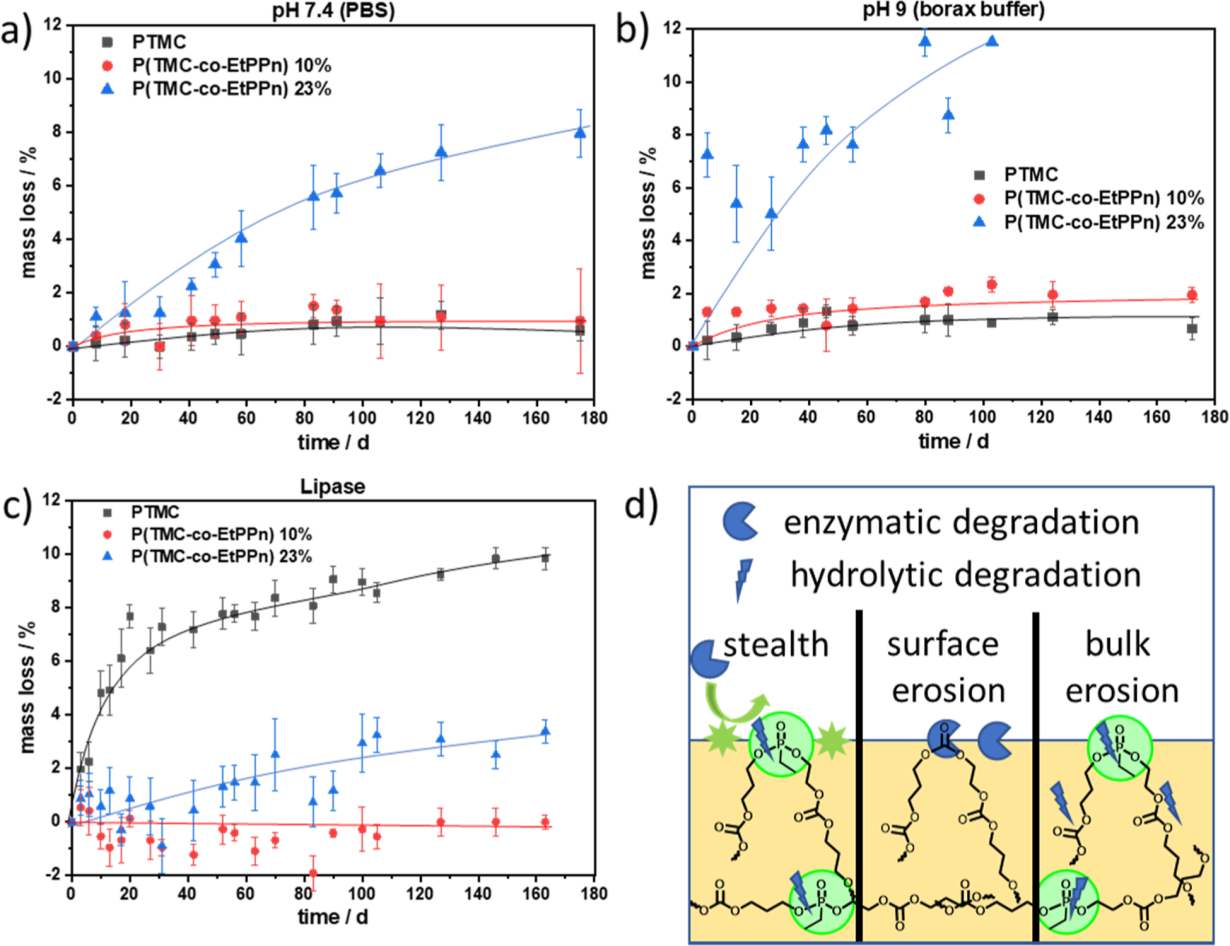
Kinetics of the abiotic and enzymatic hydrolysis of PTMC and P(TMC-*co*-EtPPn) copolymers under different conditions, (a) in PBS at pH 7.4 and (b) in borax buffer at pH 9, (c) enzyme-catalyzed hydrolysis in PBS with lipase, and (d) schematic illustration of the hydrolysis mechanism of different PTMC-based materials: PTMC degrades by surface-erosion, with increasing PPE-comonomer ratio, a bulk erosion mechanism is detected under abiotic hydrolysis, while during enzyme-treatment, the PPE-units provide stealth protection against enzymatic attack.

The enzymatic hydrolysis of polymers **1**–**3** was investigated at pH = 7.4 in the presence of 1000 U lipase from porcine pancreas at 37 °C over a period of up to 265 days (Figure 6). When the films prepared from the PTMC-homopolymer were immersed in the enzyme-containing solution, more than 10% mass loss within 150 days was detected. Surface erosion usually requires a linear mass loss; in our case the films were not stable in shape during the degradation, so the surface changed and due to shrinking the accessible surface became smaller. However, from the GPC traces we can clearly conclude that PTMC degrades mainly by surface erosion under these conditions, as the molar mass distribution did not shift to higher elution times (remaining at ca. 15 mL), and only after 27 days, a low-molecular-weight fraction started to appear at around 20 mL elution volume (Figures 5c and S4c). In contrast to the homopolymer, the films prepared from copolymers with 10 and 23 mol % EtPPn remained visually untouched over 146 days, indicating a slow enzyme attack to the more hydrophilic polymers, which might be attributed to the stealth effect, as seen by the cell repulsion discussed above (enzyme repulsion is likely that case as well). Therefore, only hydrolytic degradation occurs; as observed before, the hydrolytic degradation at pH 7.4 is only visible for P(TMC-*co*-EtPPn) 20% and can be easily followed by GPC (Figures 5b and S6c). This means by only introducing 10% EtPPn into PTMC the resulting material is switching from enzymatic degradation to very slow hydrolytic degradation following a bulk erosion mechanism for higher amounts of EtPPn (Figure 6).

## Conclusions

Rubber-like P(TMC-*co*-EtPPn) copolymers were synthesized by statistical ring-opening copolymerization of cyclic TMC and EtPPn followed by transesterification to randomize the structures. Copolymers with 10 and 23 mol % of EtPPn were prepared; while the 10 mol % EtPPn-containing copolymer showed similar mechanical properties as PTMC with comparable molar mass, the 23 mol % incorporation of EtPPn weakened the mechanical properties. In contrast to PTMC, copolymers with 10 mol % of EtPPn exhibited significant cell repulsion and did not degrade in the presence of lipase. Human mesenchymal stem cells could be cultured on PTMC and cell growth was observed within 7 days, while EtPPn-containing copolymers exhibited a stealth/antifouling effect. This stealth-like behavior also impeded enzymatic degradation by lipase, while PTMC was degraded by lipase via 10% surface erosion within 150 days. The EtPPn-containing polymers showed hydrolytic degradation with the speed depending on the amount of EtPPn in the polymer material. For high EtPPn content (>20%) a relatively fast bulk erosion was observed, while for 10% EtPPn the degradation in PBS at pH 7.4 was almost neglectable. In conclusion, PTMC polymers were modified by random copolymerization with EtPPn and the degradation mechanism was switched from enzymatic to adjustable abiotic hydrolysis, explained by the stealth properties of the hydrophilic phosphonates.

## Experimental Materials and Methods

### Materials

All solvents were purchased in HPLC grade or dry (purity >99.8%) and chemicals were purchased in the highest grade (purity >98%) from Sigma-Aldrich, Acros Organics, Fluka, or Fisher Scientific and used as received unless otherwise described. 2-Ethyl-2-oxo-1,3,2-dioxaphospholane (EtPPn) was synthesized according to the two-step procedure described by Wolf et al.^29^ The monomer was stored at −25 °C under a nitrogen atmosphere and was freshly distilled before use. Trimethylene carbonate (TMC) was provided by Huizhou Foryou Medical Devices Co., Ltd. (China) and used as received. Dulbecco’s phosphate-buffered saline (DPBS), Dulbecco’s modified Eagle’s medium (DMEM), fetal bovine serum (FBS), glutamax, trypsin/EDTA, and penicillin/streptomycin were obtained from Gibco. Gelatin solution (type B, 2% (w/v) in water, tissue culture grade), calcein-AM, lipase from porcine pancreas (>125 U/mg), and ethidium homodimer I were purchased from Sigma-Aldrich.

#### Nuclear Magnetic Resonance (NMR) Spectroscopy

^1^H, ^13^C, and ^31^P NMR spectra were measured on a 400 MHz Bruker AVANCE III AMX system or 600 MHz Bruker AVANCE NEO system. The temperature was kept at 25 °C during the measurements. As deuterated solvent, CDCl_3_ was used. MestReNova 9 from Mestrelab Research S.L. was used for analysis of all measured spectra. The spectra were calibrated against the solvent signal (CDCl_3_: δH = 7.26 ppm).

For real-time ^1^H NMR measurements in CH_2_Cl_2_ a D1 time of 10 s (8 scans) was used and for ^31^P NMR measurements a D1 time of 30 s (8 scans) was used. For real-time ^1^H NMR measurements in bulk a D1 time of 15 or 20 s (4 scans) (T1 for all components were determined <6 s) was used and for ^31^P NMR measurements a D1 time of 30 or 36 s (4 scans) (T1 for the EtPPn monomer 7.1 s and in the polymer 2.3 s) was used.

#### Gel Permeation Chromatography (GPC)

GPC measurements were performed in DMF at 50 °C with an Agilent Technologies 1260 Infinity PSS SECcurity system at a flow rate of 1 mL min^–1^. Each sample injection volume was 50 μL, and SDV columns (PSS) with dimensions of 300 × 80 mm^2^, 10 μm particle size, and pore sizes of 10^6^, 10^4^, and 500 Å were employed. Calibration was carried out using polystyrene standards supplied by Polymer Standards Service. The GPC data were plotted using the software OriginPro 8G from OriginLab Corporation.

#### Differential Scanning Calorimetry (DSC)

DSC measurements were performed using a Trios DSC 25 series thermal analysis system with the temperature range from −100 to 35 °C under nitrogen with a heating rate of 10 °C min-1. All glass transition temperatures (*T*_g_) were obtained from the second heating ramp of the experiment.

#### Tensile Tests

All mechanical tests were performed in triplicate on solvent cast films with dimensions in accordance to ASTM standard D882-91 specifications (100 × 5 × 0.1 mm^3^). The mechanical tests were carried out on a Zwick Z020 universal tensile testing machine (Germany) equipped with a 500 N load cell at room temperature (∼21 °C). The E-Modulus was derived from 0.25 to 2% strain and the crosshead speed was set to 50 mm/min for the first 2% of elongation. Tensile tests were carried out while operated at a crosshead speed of 500 mm/min. Specimen deformation was derived from the grip-to-grip separation, the initial grip-to-grip separation being 50 mm.

#### Contact Angle

Contact angle measurements were done on an optical contact angle system from DataPhysics Germany (OCA 25) running with SCA20 software. The measurements were performed at room temperature (∼21 °C). 1 mL of ultrapure water (Milli-Q, Millipore) was dispensed on solvent cast films and a picture for analysis was taken 2 s after droplet deposition. Angles were measured on different regions of each polymer surface, and the results were averaged.

### Syntheses

#### Copolymerization of EtPPn and TMC in the Bulk

In a flame-dried Schlenk tube, TMC (9.7 g, 95 mmol), EtPPn (1.42 g, 10 mmol), and Sn(Oct)_2_ (6.7 mg, 0.016 mmol) were dissolved in anhydrous benzene (10 mL) and dried by lyophilization two times, the second time overnight. The monomer mixture was placed in a preheated oil bath at 130 °C and stirring was applied but stopped after 2 h since the viscosity increased fast. The reaction was stopped after 26 h by cooling to room temperature. The polymer was dissolved in ca. 150 mL of DCM and precipitated into cold diethyl ether (ca. −5 °C, 900 mL). Then, the polymer was dried in vacuo overnight, and the pure copolymer was obtained as a colorless rubber-like material.

#### Film Preparation

Films were prepared by casting degassed polymer solutions (10–15 wt %) in chloroform on glass plates; the solvent was evaporated under dry nitrogen purging. The films were dried under reduced pressure at room temperature.

Tensile strips with the specifications of 100 × 5 × 0.2 mm^3^ were cut out from these cast films. For the cell interaction and degradation studies discs with a diameter of 10, 12, or 15 mm were punched out of these films.

#### Abiotic Hydrolysis in PBS

For hydrolysis tests a 0.01 M phosphate-buffered saline (PBS) pH 7.4 solution with sodium azide (0.02 wt/vol %) was prepared. From the prepared polymer films, discs with diameters of 15 mm were punched out and were placed on a coverslip (*d* = 25 mm). The coverslip was placed in a 6-well plate and incubated in 5 mL of PBS per well at 37 °C. At selected time points the foils were rinsed with water, dried to constant weight, and the mass loss was determined (at least triplicates were used). One extra sample were used for GPC analysis.

#### Abiotic Hydrolysis in Borax Buffer

For hydrolysis tests a 0.05 M borax buffer pH 9 solution was prepared. From the prepared polymer films, discs with diameters of 15 mm were punched out and were placed on a coverslip (*d* = 25 mm). The coverslips were placed in a 6-well plate and incubated in 5 mL of buffer per well at 37 °C. At selected time points the foils were rinsed with water, dried to constant weight, and the mass loss was determined (at least triplicates were used). One extra sample were used for GPC analysis.

#### Enzymatic Hydrolysis with Lipase

A fresh lipase solution was prepared (∼500 U/mL) containing 160 mg of lipase (from porcine pancreas), 5 mL of propylene glycol, and 35 mL of PBS (0.01 M, pH 7.4). From the prepared polymer films, discs with a diameter of 12 mm were punched out and were placed on a coverslip (*d* = 18 mm). The coverslips were placed in a 12-well plate and incubated in 2 mL of lipase solution (∼1000 U per well) at 37 °C. At selected time points the foils were rinsed with water twice, dried to constant weight, and the mass loss was determined (at least triplicates were used). One extra sample were used for GPC analysis.

#### Cell Culturing on the Polymer Films

Human mesenchymal stem cells (hMSCs, passage 5) were cultured at 37 °C in humidified air containing 5 vol % CO_2_, in 75 cm^2^ cell culture flasks containing culture medium consisting of DMEM, 1% (v/v) glutamax, 10% (v/v) FBS, and 1% (v/v) penicillin/streptomycin. The culture flasks were coated with 0.1% (v/v) gelatin solution in sterile water before cell seeding. The medium was refreshed three times per week until the cells reached confluence. Upon confluence, the cells were trypsinized and counted using an EVE automated cell counter. Polymer disks of the three polymer samples with a diameter of 10 mm and a thickness of 0.1 mm were placed in a 48-wells suspension culture plate (not surface-treated for cell culturing). Subsequently, the polymer foils were disinfected with 70% (v/v) ethanol in water for 10 min, washed twice with DPBS, and kept in cell culture medium overnight at 37 °C. The hMSCs were seeded on the networks at a density of 8000 cells per well and cultured for 7 d. The medium was refreshed three times per week.

Live/dead staining was performed on days 1, 4, and 7 after cell seeding. The polymer discs were rinsed with warm DPBS (37 °C) and incubated with 2 μM calcein-AM/4 μM ethidium homodimer-1 solution in culture medium for 1 h. After rinsing with warm DPBS, pictures were taken using a Olympus IX71 fluorescent microscope with the Olympus cellSens Dimension software.

## Data Availability

The raw data are available upon request.

## Supporting Information Available

Additional characterization data, such as GPC, NMR, photos, and mechanical properties, and detailed plots of the degradation (PDF)

## Notes

There are no clinical or animal studies associated with this work.
