# Supplementary material for: Rubber-like and Antifouling Poly(trimethylene carbonate-ethylphosphonate) Copolymers with Tunable Hydrolysis
Source: ACS Appl Mater Interfaces. 2025 Apr 15;17(16):23513–21. doi: 10.1021/acsami.4c21079 (PMC12022945; doi:10.1021/acsami.4c21079)
Supplement: Supplementary file 1 — am4c21079_si_001.pdf [file am4c21079_si_001.pdf]

Supporting Information

Rubber-like and Antifouling Poly(trimethylene carbonate-ethylphosphonate) Copolymers with Tunable Hydrolysis

*Timo Rheinberger,[a] Marc J. K. Ankone,[b] Dirk W. Grijpma,[b] Frederik R. Wurm[a]**

a) Sustainable Polymer Chemistry (SPC), Department of Molecules and Materials, MESA+ Institute for Nanotechnology, Faculty of Science and Technology, University of Twente, P.O. Box 217, 7500 AE Enschede (Netherlands), f.r.wurm@utwente.nl

b) Department of Advanced Organ bioengineering and Therapeutics (AOT), Faculty of Science and Technology, University of Twente, Enschede, the Netherlands

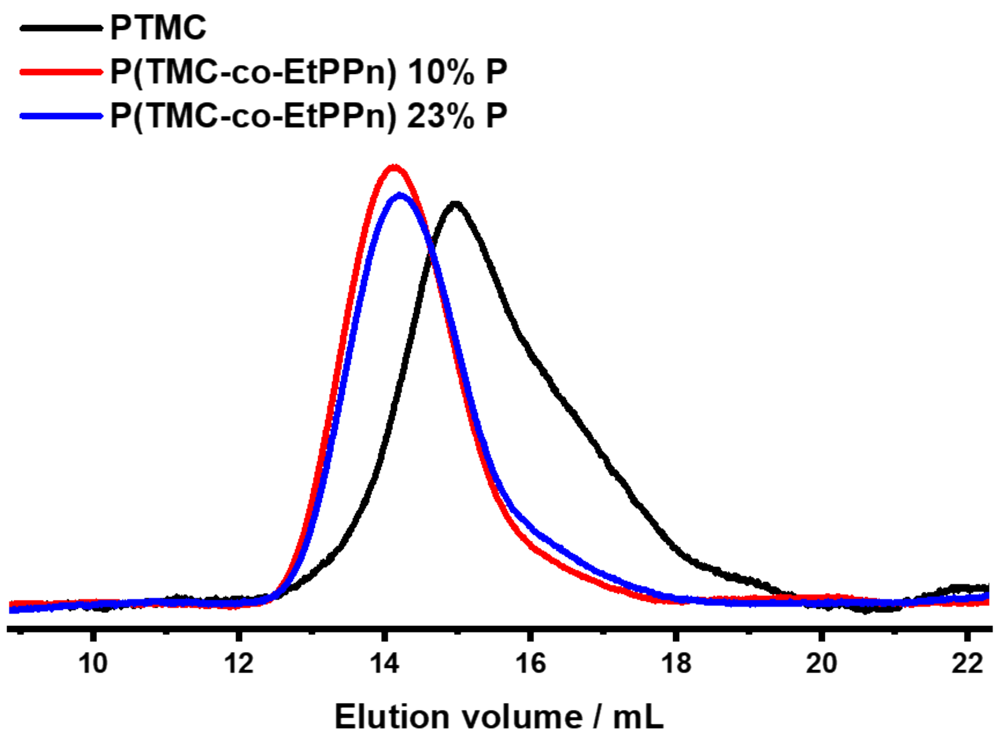

Figure S1: GPC traces of the synthesised polymers **1a, 2,** and **3** (measured in DMF with 0.1 mol · L^{-1} LiCl).

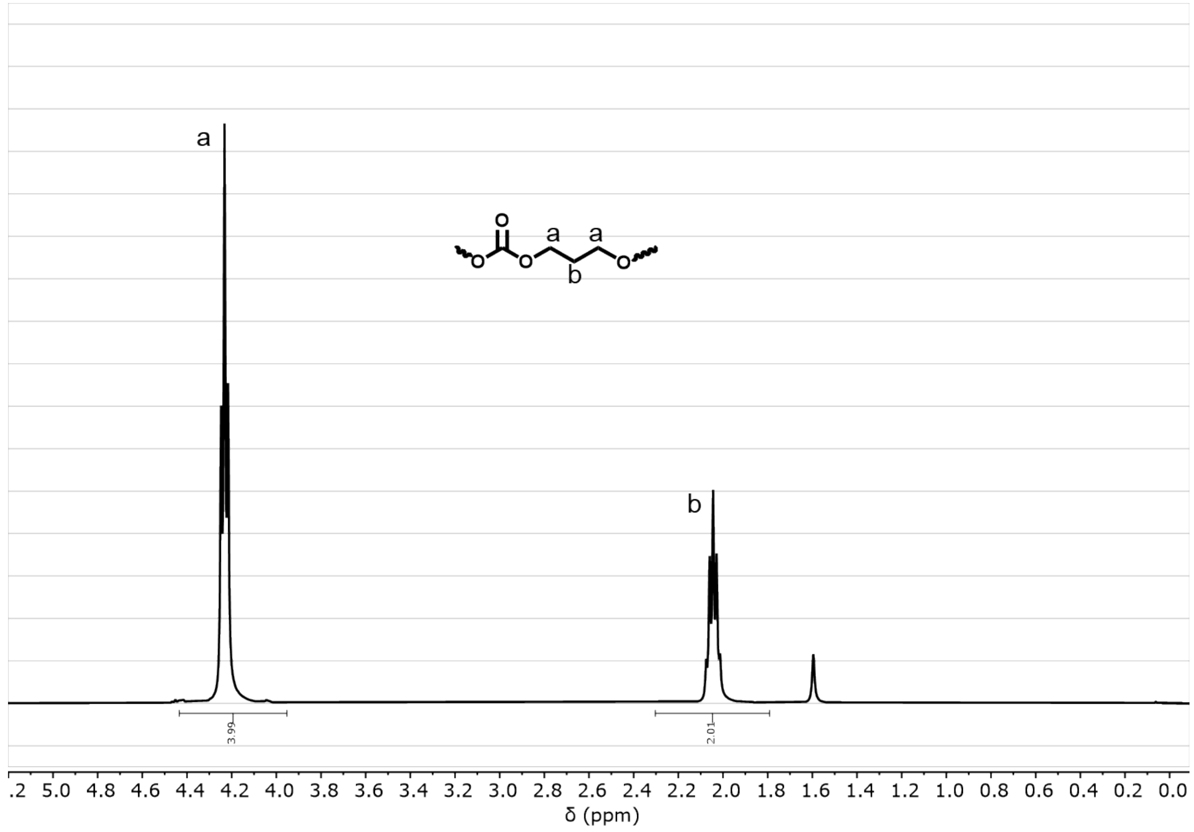

Figure S2: ^{1}H NMR (400 MHz, 298 K, $CDCl_3$) spectrum of polymer **1a** P(TMC) polymerised in bulk at 130 °C.

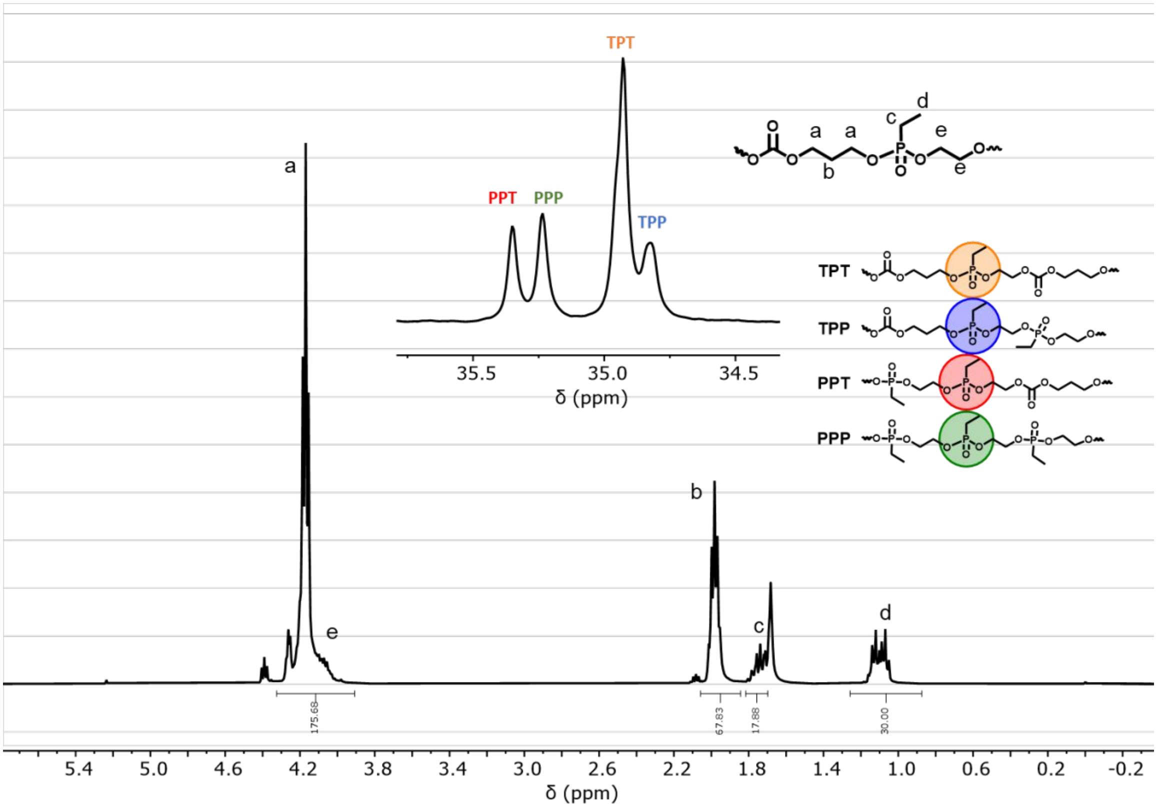

Figure S3: ^{1}H NMR (400 MHz, 298 K, $CDCl_3$) and inset $^{31}P\{H\}$ NMR (161 MHz, 298 K, CDCl3) spectra of polymer **3** P(TMC-*co*-EtPPn) 23 % polymerised in bulk at 130 °C.

Table S1: Summarized mechanical properties of synthesised copolymers P(TMC-*co*-EtPPn).

#	Polymer	M_n / kg · mol^{-1} [a]	h / dL · g^{-1}	E-Modulus[b] / MPa	s_{yield} [b] / MPa	e_{yield} [b] / %	s_{break} [b] / MPa	e_{break} [b] / %
**1a**	PTMC	90	-	3.2 ± 0.2	1.00 ± 0.01	105 ± 10	0.44 ± 0.05	620 ± 90
**1b**	PTMC[c]	273[c]	4.6[c]	6.2[c]	2.3[c]	140[c]	16[c]	850[c]
**2**	P(TMC-*co*-EtPPn) 10 %	218	2.6	4.3 ± 0.3	1.62 ± 0.01	150 ±	0.95 ± 0.02	700 ± 30
**3**	P(TMC-*co*-EtPPn) 20 %	175	2.0	2.8 ± 0.1	1.10 ± 0.06	140 ± 10	0.9 ± 0.1	200 ± 20

a) determined from gel permeation chromatography (GPC) measurements, b) determined from stress strain measurements on a tensile testing machine, (results are shown as mean and standard error of the mean. c) homo PTMC data from Pêgo *et al*..[20]

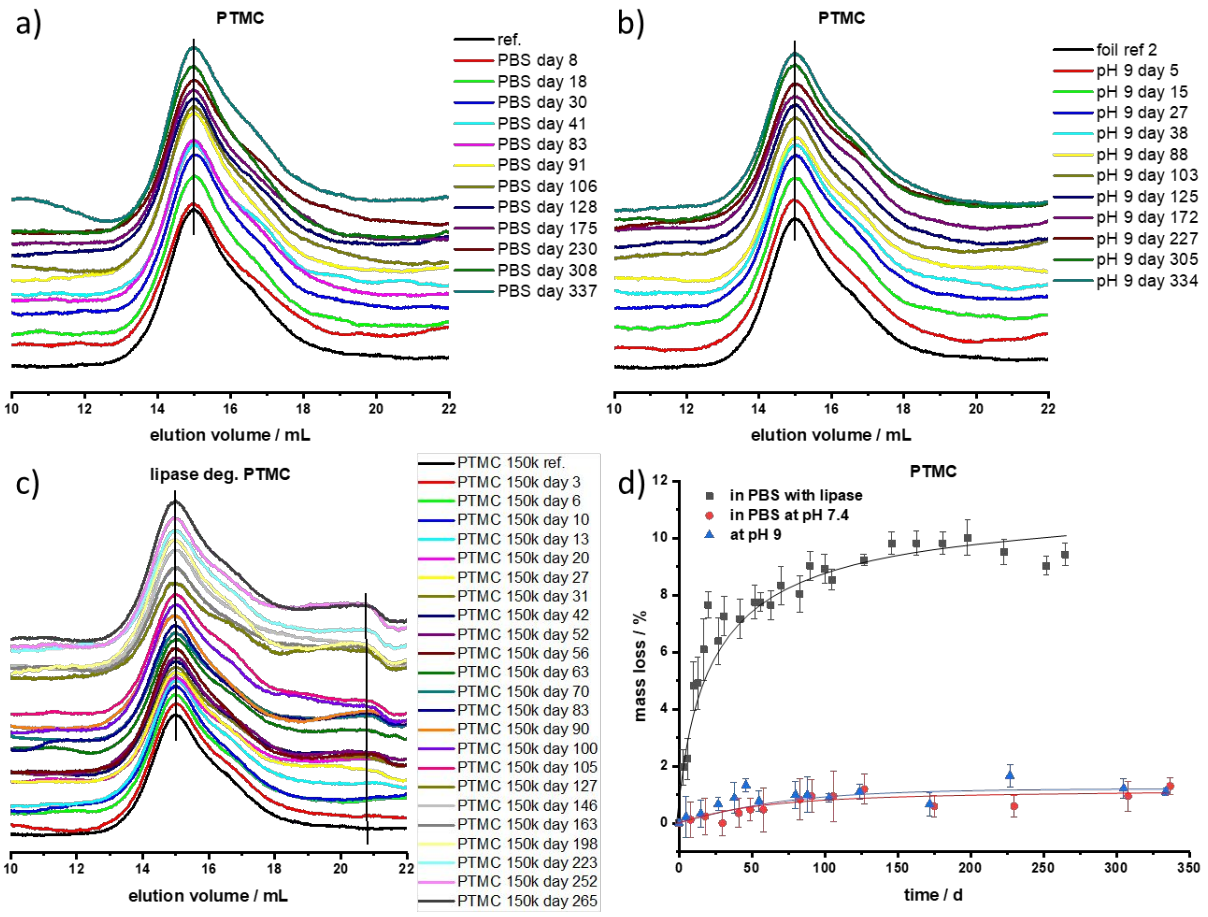

Figure S4: Summarised data from all hydrolysis experiments of PTMC (1a), GPC traces from polymer film samples after different degradation time points under different conditions a) in PBS at pH 7.4, b) at pH 9 and c) in PBS with lipase; d) shows the degradation caused mass loss for all three hydrolysis conditions.

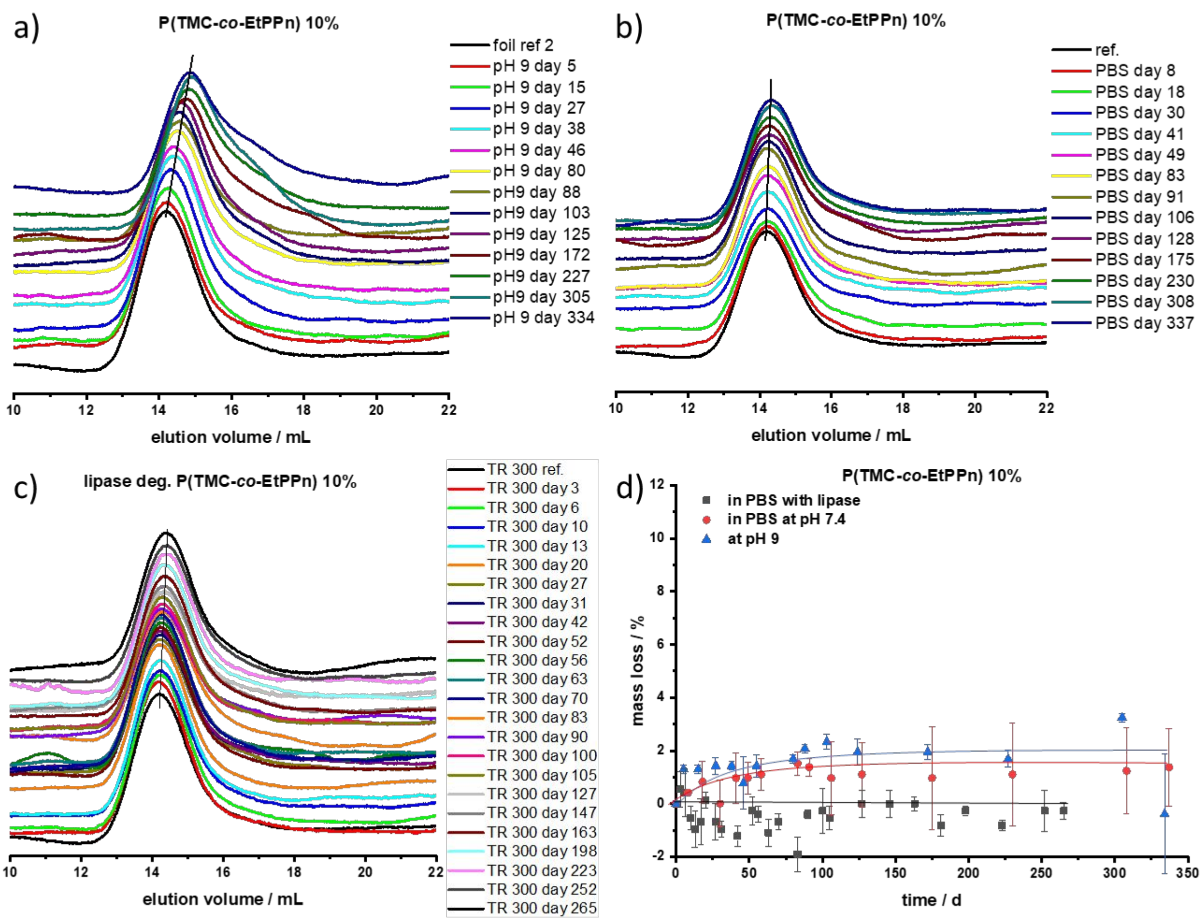

Figure S5: Summarised data from all hydrolysis experiments of P(TMC-*co*-EtPPn) 10 %, GPC traces from polymer film samples after different degradation time points under different conditions a) in PBS at pH 7.4, b) at pH 9 and c) in PBS with lipase; d) shows the degradation caused mass loss for all three hydrolysis conditions.

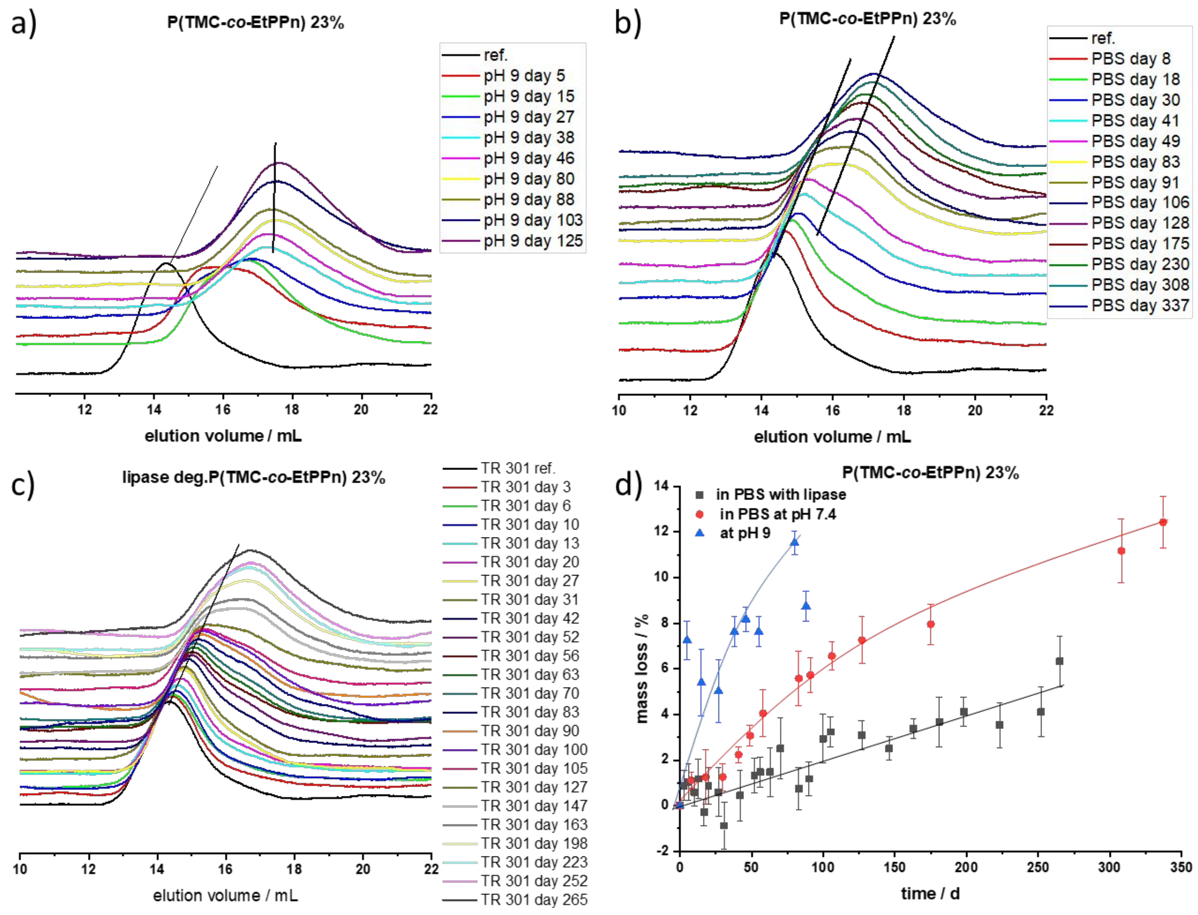

Figure S6: Summarised data from all hydrolysis experiments of P(TMC-*co*-EtPPn) 23 %, GPC traces from polymer film samples after different degradation time points under different conditions a) in PBS at pH 7.4, b) at pH 9 and c) in PBS with lipase; d) shows the degradation caused mass loss for all three hydrolysis conditions.

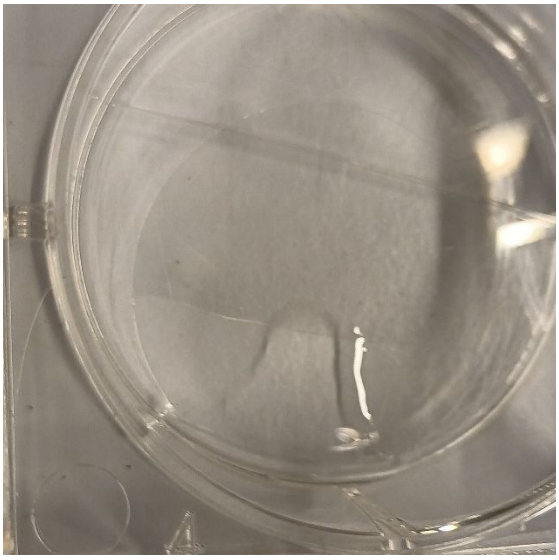

Figure S 7 Polymer film hydrolysed into a liquid droplet floated from the coverslip, polymer **3** after 125 days at pH 9.
